# Real-time ultrasound-guided percutaneous dilatational tracheostomy: a feasibility study

**DOI:** 10.1186/cc10047

**Published:** 2011-02-22

**Authors:** Venkatakrishna Rajajee, Jeffrey J Fletcher, Lauryn R Rochlen, Teresa L Jacobs

**Affiliations:** 1 Departments of Neurosurgery and Neurology, University of Michigan Health System, 3552 Taubman Health Care Center, 1500 E. Medical Center Dr., SPC 5338, Ann Arbor, MI 48109-5338, USA; 2 Department of Anesthesiology, University of Michigan Health System, University Hospital, 1500 E. Medical Center Drive, Room 1H247, Ann Arbor, MI 48109-5048, USA

## Abstract

**Introduction:**

Ultrasound (US) performed prior to percutaneous tracheostomy (PT) may be useful in avoiding injury to pretracheal vascular structures and in avoiding high placement of the tube. Bedside real-time US guidance with visualization of needle path is routinely utilized for other procedures such as central venous catheterization, and may enhance the safety and accuracy of PT without causing airway occlusion or hypercarbia. Our objective was to demonstrate that PT performed under real-time US guidance with visualization of needle path during tracheal puncture is feasible, including in patients with features that increase the technical difficulty of PT.

**Methods:**

Mechanically ventilated patients with acute brain injury requiring tracheostomy underwent US guided PT. The orotracheal tube was withdrawn using direct laryngoscopy. The trachea was punctured under real-time US guidance (with visualization of the needle path) while using the acoustic shadows of the cricoid and the tracheal rings to identify the level of puncture. After guidewire passage the site and level of entry was verified using the bronchoscope, which was then withdrawn. Following dilatation and tube placement, placement in the airway was confirmed using auscultation and the "lung sliding" sign on US. Bronchoscopy and chest X-ray were then performed to identify any complications.

**Results:**

Thirteen patients successfully underwent US guided PT. Three patients were morbidly obese, two were in cervical spine precautions and one had a previous tracheostomy. In all 13 patients bronchoscopy confirmed that guidewire entry was through the anterior wall and between the first and fifth tracheal rings. There was no case of tube misplacement, pneumothorax, posterior wall injury, significant bleeding or other complication during the procedure.

**Conclusions:**

Percutaneous tracheostomy performed under real-time ultrasound guidance is feasible and appears accurate and safe, including in patients with morbid obesity and cervical spine precautions. Larger studies are required to further define the safety and relative benefits of this technique.

**Trial registration:**

UMIN Clinical Trials Registry, UMIN000005023.

## Introduction

Percutaneous tracheostomy (PT) is now a commonly performed bedside procedure in the Intensive Care Unit (ICU). Several studies have demonstrated that PT is a safe and cost-effective alternative to open, surgical tracheostomy [1-3]. Bronchoscopic guidance during PT may be useful in avoiding injury to surrounding structures, high placement of the tube, injury to the posterior tracheal wall and in confirming endotracheal placement [4,5]. The use of bronchoscopy, however, requires the availability of specialized equipment, staff and specific expertise. In patients with acute brain injury, acute elevations in intracranial pressure may occur during the performance of bronchoscopy [6].

Preliminary reports suggest that sonographic delineation of anatomy prior to tracheal puncture during PT may help prevent bleeding from pretracheal vascular structures and placement of the tracheal tube above the first tracheal ring [7-9]. The use of real-time ultrasonography, with actual visualization of the needle path up to the anterior tracheal wall should further decrease the risk of puncture above the first tracheal ring as well as the risk of injury to surrounding structures and the posterior tracheal wall. While the use of real-time sonographic imaging with visualization of the needle path is routinely used for other bedside procedures, such as the insertion of central venous catheters [10,11], real-time sonographic guidance of the needle path during PT has not yet been described in the literature. Real-time guidance during PT may be particularly useful when factors that increase the technical difficulty of the procedure (morbid obesity, difficult anatomy, cervical spine precautions) are present. Ultrasound imaging may permit accurate delineation of the position of the tracheal rings prior to puncture in these patients despite the absence of clearly palpable tracheal anatomy (in patients with morbid obesity) and without extending the neck (in patients with cervical spine precautions). Our objective was to demonstrate that PT performed under real-time US guidance with visualization of needle path during tracheal puncture is feasible, including in patients with features that increase the technical difficulty of PT.

## Materials and methods

Approval was obtained from the Institutional Review Board of the University of Michigan for this study. Consecutive patients in the neuro-intensive care unit of the University of Michigan scheduled to undergo bedside tracheostomy between May and November 2010 were prospectively enrolled to undergo ultrasound guided percutaneous tracheostomy (US-PT) based on consent and investigator availability. Consent was obtained from next of kin. Initial sonographic examination of anatomy was performed after consent was obtained. Following consent, criteria for performing PT under standard bronchoscopic guidance rather than US-PT were: 1) the inability to clearly visualize the first tracheal ring above the sternal notch on ultrasound and 2) the inability to obtain at least a Cormack-Lehane Grade 2b view (view of the arytenoids) on direct laryngoscopic examination. All US-PTs were performed by a single intensivist (VR) with six years' experience performing PT and three years' experience with the use of point-of-care ultrasound for evaluation of anatomy prior to PT.

### Timing of and indications for tracheostomy

The decision to perform tracheostomy was made in accordance with the usual practice at our institution. The number of days on mechanical ventilation prior to PT, and the indication for tracheostomy were recorded. Cervical spine precautions, sub-optimal anatomy to palpation, obesity (Body Mass Index, BMI, ≥30 kg/m^2^) and previous tracheostomy were not considered to be automatic contra-indications to PT, in accordance with previously published studies that have demonstrated the safety of this technique in these groups of patients [12-14].

### Percutaneous tracheostomy technique

**Figure 1:**
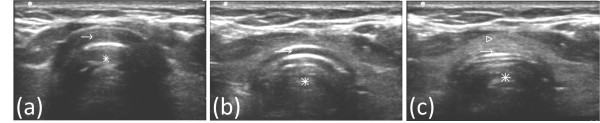
**Axial images of trachea and pretracheal structures on ultrasound**. *Asterisk*: Tracheal lumen. **(a) ***Arrow*- acoustic shadow of cricoid cartilage. **(b) ***Arrow*- acoustic shadow of first tracheal ring. **(c) ***Arrow: *Anterior tracheal wall between first and second tracheal rings. *Arrowhead*- Thyroid isthmus.

**Figure 2:**
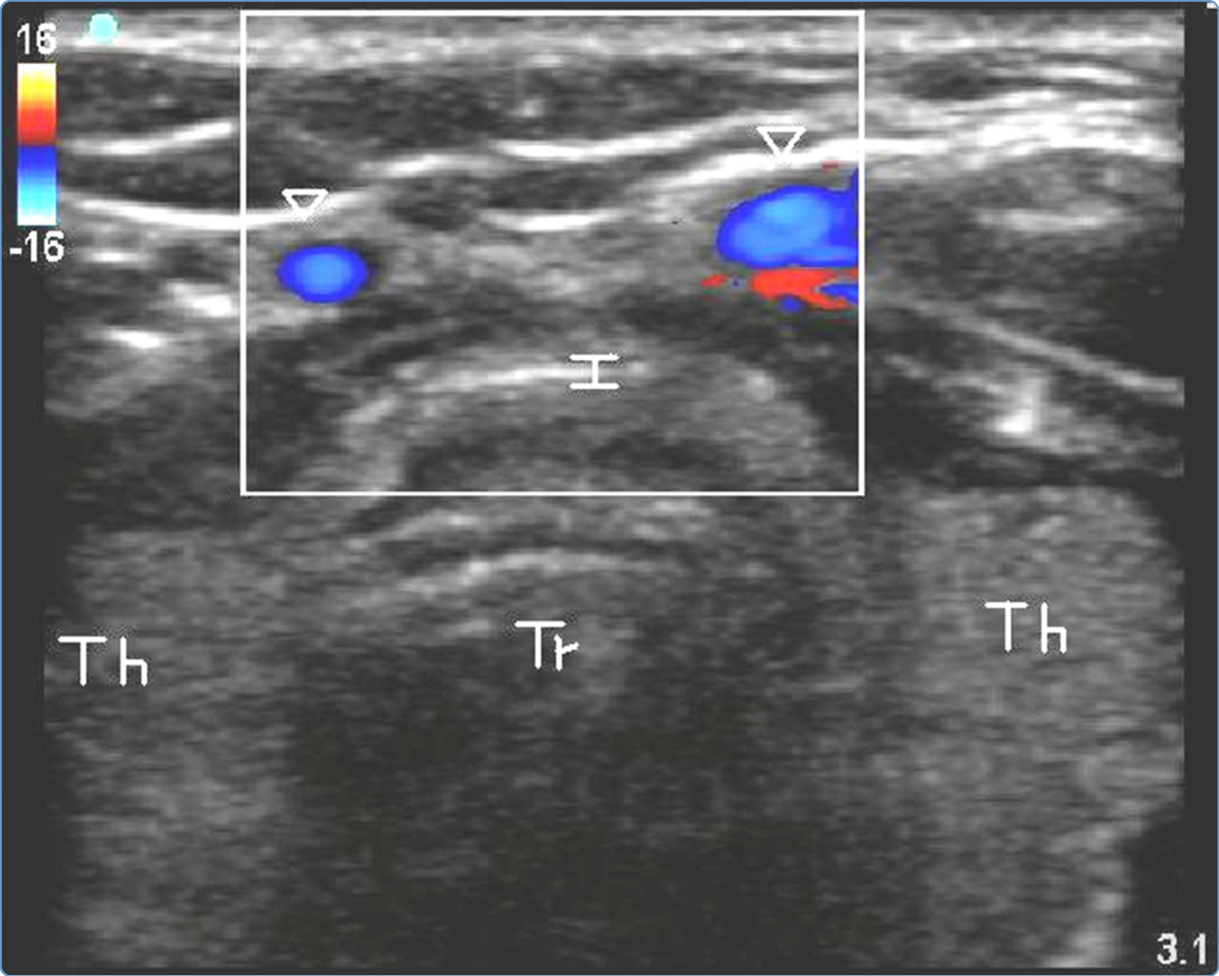
**Axial image of trachea and surrounding structures with depiction of pre-tracheal veins using color duplex imaging**. *Tr*, Tracheal lumen; *Th*, lobes of thyroid; *I*, thyroid isthmus; *Arrowheads*, pre-tracheal veins.

**Figure 3:**
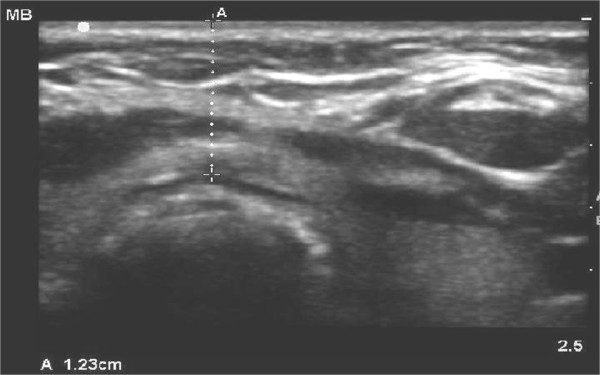
**Measurement of skin to anterior tracheal wall thickness at the level of the second tracheal ring**. Measured distance is 1.23 cm.

Tracheal and pre-tracheal anatomy was examined using palpation as well as the ultrasound (Figures 1a-c and 2) after enrollment for US-PT. The ultrasound was used to confirm that the first tracheal ring was clearly visible above the sternal notch with the neck in the anticipated position for the tracheostomy (extension for most patients, neutral position for patients with cervical spine precautions). For morbidly obese patients the ultrasound was used to estimate the thickness of soft tissue between the skin and the trachea at the level of the second tracheal ring (Figure 3), as well as the internal diameter of the trachea itself at that level, with the head in the neutral position, in order to assess the need for an extended-length tracheostomy tube and the most appropriate size of tracheostomy tube. The use of skin to trachea sonographic measurements to determine appropriate tracheostomy tube length has been previously described [15].

A propofol infusion was used for all patients for the duration of the procedure (both tracheostomy and subsequent bronchoscopy), titrated to deep sedation (Richmond Agitation Sedation Score of -5) prior to administration of the neuromuscular blockade. Fentanyl and vecuronium were administered to all patients prior to commencement of the procedure. Following induction, the endotracheal tube was withdrawn under direct laryngoscopic vision until the cuff was positioned immediately inferior to the vocal cords. Standard Macintosh and Miller laryngoscope blades of the appropriate size were used. The Cook Ciaglia Blue Rhino^® ^G2 (Cook Medical Inc., Bloomington, IN, USA) single stage dilator percutaneous tracheostomy kit was used. Continuous monitoring of heart rate, blood pressure and pulse oximetry was performed. Intracranial pressure (ICP) was monitored in patients with external ventricular drains (Bactiseal^® ^catheters, Codman & Shurtleff Inc., Raynham, MA, USA) or intraparenchymal probes (Codman^® ^MicroSensor, Codman & Shurtleff Inc., Raynham, MA, USA) in place. All ICP elevations to >25 mmHg as well as the peak ICP during the procedure were recorded, along with the stage of the procedure during which elevations and peak ICP were noticed.

**Figure 4:**
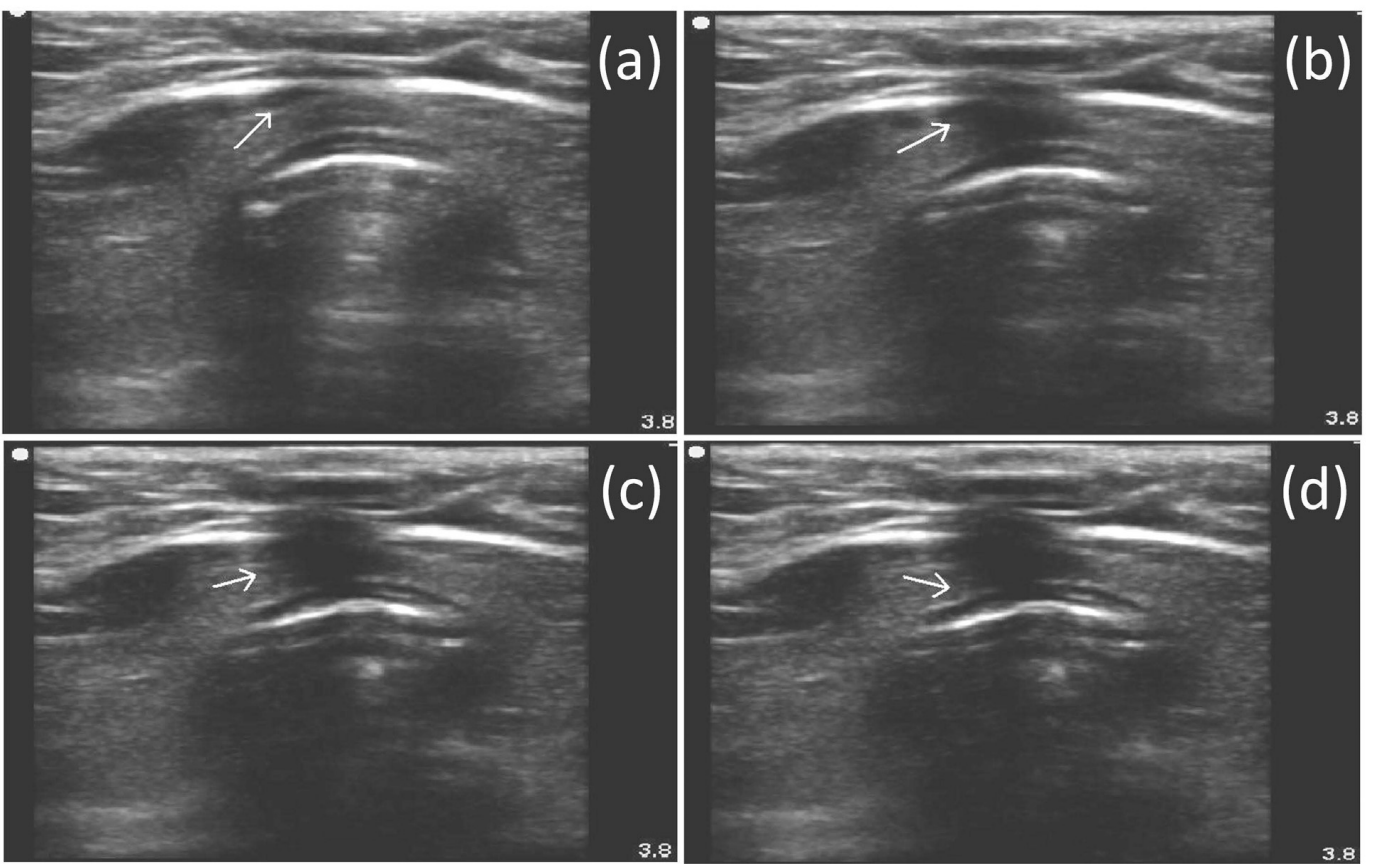
**Acoustic shadow (*Arrow*) and displacement of tissue depicting the path of the needle during tracheal puncture**.

A Sonosite M-Turbo^® ^(SonoSite Inc., Bothell, WA, USA) point-of-care ultrasound machine was used, with a 10 to 5 MHz linear array probe and a sterile sheath. The mode of imaging was set to maximal resolution and depth of imaging adjusted to keep the trachea just within the screen. Transverse/axial (rather than longitudinal/sagittal) real-time imaging of the trachea was performed to permit clear visualization of the needle path up to the midline of the anterior wall of the trachea. On axial imaging, the airway in the neck is immediately apparent in the midline with mixed hyper-echogenecity within the air-filled lumen. The cricoid cartilage (Figure 1a) was identified using its relatively larger acoustic shadow within the anterior wall of the larynx caudal to the cricothyroid membrane and the tracheal rings identified by their relatively thin acoustic shadows within the anterior wall of the trachea (Figure 1b). The thyroid gland and isthmus were delineated (Figures 1c and 2). The point of tracheal puncture was selected using the following criteria on sonographic imaging: below the first tracheal ring but above the fifth tracheal ring and no vascular structure (Figure 2) in the path of the needle. Ideally, the space between the second and third rings or the third and fourth tracheal rings was selected; however, the precise inter-tracheal ring space was considered less important than passage below the first and above the fifth tracheal rings. Puncture through the thyroid isthmus was permitted. The 15 G needle was introduced perpendicularly to the skin and the needle path was determined by the distinct acoustic shadow ahead of the needle followed by the displacement of tissue layers seen with needle passage (Figure 4a-d). Indentation of the anterior tracheal wall by the needle was then sometimes visible. Advancement of the needle was halted when the needle was seen to reach and then just pass the anterior wall, with a palpable change in resistance as the lumen was entered. The goal was to puncture the anterior quadrant of the trachea, as close as possible to the midline, as is our practice with standard bronchoscopic guidance. Endotracheal position of the tip was confirmed by the aspiration of air into a saline-filled syringe. The needle was then angled slightly caudally to prevent retrograde passage of the guidewire. The guidewire was then introduced and the needle removed. The bronchoscope was then passed through the endotracheal tube, the exact point of guidewire entry recorded and the trachea visualized for any sign of injury or posterior wall puncture. The bronchoscope was then removed. A 2 cm horizontal incision was made at the point of guidewire entry and blunt dissection was carried out. The 14Fr dilator was then used to create the initial stoma, followed by the single-stage "Rhino Horn" dilator over the guidewire and guiding catheter. The appropriate-sized tracheostomy tube fitted over an appropriate loading tube was then passed through the stoma and secured. Endotracheal placement of the tube was confirmed immediately using auscultation, verification of appropriate breath delivery on the ventilator and the presence of the sonographic "lung-sliding" bilaterally, as previously described. The lung sliding sign is the visible "sliding" of the visceral pleura on the parietal pleura on ultrasound imaging through the intercostal space along with a characteristic appearance on M-mode [16,17]. When seen bilaterally with each delivered breath through the tracheal tube, this sign denotes bilateral lung expansion.

The bronchoscope was then re-introduced through the tracheostomy tube as well as the oro-tracheal tube to look for any complications, such as airway injury, tube misplacement or tracheal ring fracture. A chest X-ray was obtained on all patients to look for further complications, such as pneumothorax or pneumo-mediastinum. Bronchoscopy was performed using Olympus BF-1T30, BF-1T40 and BF-P40 fiber-optic bronchoscopes with an Olympus Evis Exera 2 video system (Olympus America, Center Valley, PA, USA).

## Results

A total of 13 patients underwent US-PT. Sonographic delineation of anatomy was possible in all enrolled subjects and no patients required conversion to standard bronchoscopic PT. There were nine women and four men, with a mean age of 46 years (standard deviation 15 years, range 20 to 68 years). The median BMI was 28.4 kg/m^2 ^(95% central range: 19.3 to 62.5 kg/m^2^). Diagnoses were: aneurysmal subarachnoid hemorrhage (SAH, *n *= 4), severe traumatic brain injury (TBI, *n *= 2), ischemic stroke (*n *= 2), intracerebral hemorrhage (*n *= 2), severe sepsis (*n *= 1), hepatic encephalopathy with chronic obstructive pulmonary disease (COPD) (*n *= 1) and stiff-person syndrome (*n *= 1). Tracheostomy was performed a mean of four days (SD: 3 days, range 0 to 12 days) following initiation of mechanical ventilation. Two patients required tracheostomy because of the need for prolonged mechanical ventilation. The indication for tracheostomy in the other 11 patients was poor mental status with an inability to cough effectively and clear secretions.

Two of 13 patients (including one with BMI 36 kg/m^2^) were in cervical spine precautions. One patient (with BMI 33 kg/m^2^) had had a previous tracheostomy. Six of 13 patients were obese (BMI ≥30 kg/m^2^), while three were morbidly obese (BMI ≥40 kg/m^2^). One patient with extreme obesity had a BMI of 65.9 kg/m^2^. Four patients, including all three patients with BMI >40 kg/m^2 ^and one patient in cervical spine precautions had anatomy that could not be adequately defined by palpation.

### Ultrasound findings

Tracheal anatomy could be adequately defined in all patients on ultrasound and tracheal puncture achieved with a single advance of the needle in all patients. Adequate sonographic delineation of anatomy with the linear probe was possible in all enrolled patients, regardless of BMI. The first tracheal ring was visualized above the sternal notch in all patients. Two patients were found to have midline pretracheal veins, presumed to be inferior thyroid veins, in the planned path of puncture, requiring a change in the site of puncture. The needle path could be defined using the acoustic shadow ahead of the needle followed by displacement of tissue in all patients (Figure 4a-d). In 4 of 13 (31%) patients, actual indentation of the anterior tracheal wall during tracheal puncture could be seen. In these patients, a subsequent straightening of the anterior wall was seen once the anterior wall had been breached.

Skin to trachea measurements in the three morbidly obese (BMI >40 kg/m^2^) patients were 1.23 cm (BMI 42 kg/m^2^, internal tracheal diameter, ITD, 1.34 cm), 1.4 cm (BMI 43 kg/m^2^, ITD 1.51 cm) and 2.97 cm (BMI 65.9 kg/m^2^, ITD 1.44 cm). Accordingly, the first two morbidly obese patients had standard length Shiley^® ^(Covidien-Nellcor, Boulder, CO, USA) size 8.0 tracheostomy tubes placed while the patient with BMI 66 kg/m^2 ^had an extended proximal length Tracheosoft^® ^size 7.0 tube (Covidien-Nellcor, Boulder, CO, USA) placed successfully.

### Bronchoscopic findings

Guidewire placement was through the anterior quadrant and was judged adequate in all patients on bronchoscopy. Guidewire entry was between the third and fourth tracheal rings in seven patients, second and third rings in three patients, fourth and fifth rings in two patients and first and second rings in one patient. Both patients with guidewire entry between the fourth and fifth tracheal rings had pretracheal vascular structures that were specifically avoided. No complications were found on bronchoscopy, including no clearly visible tracheal ring fractures and no posterior wall injury/puncture.

### Monitoring of physiological parameters

There were no episodes of hypoxia (pulse oximetry <90%) or significant hemodynamic instability during the performance of PT. Seven of 13 patients had ICP monitored during the procedure (2 with TBI, 2 with SAH and 1 with ischemic stroke). Of note, all but two of these patients (both of whom had undergone decompressive craniectomy) demonstrated transient (recorded as lasting for less than five minutes each time) elevations of ICP to >25 mmHg. The average maximum ICP seen during the procedure was 29 mmHg (SD: 9 mmHg, range 15 to 39 mmHg). Of note, the maximum recorded ICP during the procedure was always during bronchoscopy, although a smaller increase in ICP, for much shorter duration (recorded as being less than one minute in each instance), was also noted during direct laryngoscopy and passage of the single stage dilator and the tracheostomy tube. Retention of the bronchoscope in the airway was limited to no more than five minutes at a time, to limit ICP elevation.

### Complications and follow-up

No complications were seen on bronchoscopy or chest X-ray. The tube was seen to be in good position within the trachea in all patients on bronchoscopy and chest X-ray, with the tip positioned within the thoracic cavity and at least 2 cm above the trachea. Follow-up was available for an average period of four months (SD: three months, range one week to seven months) following tracheostomy. Three patients had died, all from withdrawal of care, of causes unrelated to tracheostomy (two for failure to demonstrate any neurological recovery and one for failure to wean from mechanical ventilation with multiple medical co-morbidities). Five patients had undergone successful decanulation of the tracheal tube, a mean of 17.6 days (SD: 4.5 days, range 12 to 24 days) from tracheostomy. One female patient on mechanical ventilation with BMI 33 kg/m^2^, adequately palpable pre-procedure neck anatomy and a standard length Shiley^® ^6.0 tube suffered dislodgment of the tracheostomy tube seven days after tracheostomy during a period of severe agitation with head shaking and developed acute respiratory distress while the tube was dislodged. The tube was emergently replaced through the stoma and the patient had no permanent injury from the accidental decanulation. The tube was subsequently empirically changed to an extended proximal length size 6.0 tube to decrease the risk of future dislodgement. No other complications, minor or major, were observed in any patient during the available period of follow-up.

## Discussion

The purpose of our study was to demonstrate the feasibility of performing percutaneous tracheostomy under real-time ultrasound guidance with actual visualization of the needle path and to assess the accuracy of this technique in placement of the guidewire below the first tracheal ring. Tracheostomy was typically performed early (mean four days after initiation of mechanical ventilation). Our practice is to perform early tracheostomy, within one week of intubation, for patients with acute brain injury, who, in the judgment of the treating clinician, are likely to require mechanical ventilation, or a definitive airway (because of poor mental status and the inability to cough or handle secretions) for more than two to three weeks. Our rationale for performing early tracheostomy, the benefits of which are a subject of debate, is a potential reduction in the number of ventilator and ICU days [18,19] as well as improvement in patient comfort and reduced need for sedation [20].

The use of real-time sonography with visualization of the needle path for central venous catheter placement is now widespread and may decrease the rate of complications [10,11]. We believe that this technique, which has not previously been described in the literature with PT, has many potential advantages over other techniques of PT. The first is the ability to consistently place the tracheostomy tube below the first tracheal ring. Placement of the tracheal tube above the first tracheal ring may increase the risk of late sub-glottic cicatrization and stenosis [21-23]. In one study of patients who underwent autopsy following PT, 5 of 15 patients had the tracheal tube placed above the first tracheal ring when the tube was placed blindly vs zero of 11 patients when PT was performed with ultrasound guidance [8]. In this study, however, demonstration of the trachea on ultrasound was in sagittal section to determine the appropriate level of puncture. Actual visualization of the needle path and, therefore, the actual level of puncture was not possible. Real-time imaging of the needle path allows visual confirmation that the anterior wall has been passed, at which point the needle is advanced no further and posterior wall injury is avoided. Although a special metal stopper was used in the aforementioned study to avoid posterior wall injury, it is custom-made and not widely available. A further strength of our study is that all guidewire and final tracheal tube positions were immediately verified with bronchsocopy, unlike previous studies with ultrasound which either did not use real-time guidance or were able to confirm tube position only in select patients who underwent autopsy.

In this limited feasibility study, the presence of morbid obesity, sub-optimal palpable neck anatomy, previous tracheostomy or cervical spine precautions did not appear to be a barrier to the performance of US-PT. Prior studies have shown that PT should not be automatically contraindicated in these groups of patients [12-14]. About half the patients in our series had one of these factors: morbid obesity in three (including one patient with BMI 65.9 kg/m^2^), cervical spine precautions in two and previous tracheostomy in one. We believe that our technique of real-time guidance will further enhance the safety and ease of performance of PT in these sub-groups. In our series, these factors appeared to present no increased difficulty for the performance of ultrasound guided puncture, as long as the first tracheal ring was clearly visible above the sternal notch. Particularly useful may be the ability to measure the pretracheal soft tissue thickness in the morbidly obese and the consequent ability to assess the need for extended-length tracheostomy tube placement, as has been described earlier [15]. The patient in our study who suffered a late dislodgement while severely agitated had not had these measurements performed as she was not morbidly obese and appeared to have well-palpable anatomy prior to the procedure. It is possible that routine measurements of pretracheal thickness, even in patients with normal palpable anatomy, may help better select the optimal tube for placement and decrease the rate of subsequent tracheostomy dislodgement [15].

Another advantage of US-PT is the ability to avoid vascular structures anterior to the trachea. Prior studies have demonstrated a potential role for pre-procedure ultrasound imaging in transverse section to identify vascular structures and reducing the risk of bleeding [7,24]. In one study, bleeding from injury to vascular structures which would have likely been identified had ultrasound been used was considered significant in 24 of 497 (5%) PTs performed without pre-procedure US evaluation, with 6 of 24 patients requiring conversion to surgical tracheostomy [25]. Pre-procedure ultrasound resulted in a change in the planned site of tracheal puncture in 24% of patients in another study [26]. These studies did not use real-time guidance. In our study, 2 of 10 patients (20%) had the planned site of puncture moved (both caudally) to avoid vascular structures. The use of real-time imaging may be preferable for avoiding vascular structures compared to pre-procedure imaging alone, since avoidance of a vascular structure such as an inferior thyroid vein cannot be taken for granted without actual visualization of the needle path.

US-PT also does not have some of the disadvantages of bronchoscopy. This may be particularly relevant to the group of patients in whom this study was performed - patients with acute brain injury. Our study confirms a previously reported observation that bronchoscopy is associated with a predictable, if transient, increase in intracranial pressure, probably caused by hypoventilation and hypercarbia [6]. This may be particularly true when a policy of performing early, rather than late, tracheostomy is used [27], as is the practice in several neuro-ICUs including ours. Although PT has been demonstrated to be safe, overall, in patients requiring ICP monitoring [28], the use of real-time ultrasound guidance minimizes hypercarbia and the consequent elevation of ICP compared to puncture under continuous bronchoscopic monitoring and, therefore, may be preferable for patients at significant risk of developing intracranial hypertension and ICP plateau waves.

The ability to perform US-PT without bronchoscopy is limited, however, by the need to safely retract the orotracheal tube to a position high enough to permit tracheal puncture while avoiding accidental extubation. We used direct laryngoscopy for this purpose, to demonstrate one potential method of safely performing US-PT without bronchoscopy. An adequate laryngoscopic grade of view was, therefore, a pre-requisite. For patients with an inadequate laryngoscopic view of the glottis, or operators who do not routinely perform direct laryngoscopy, other non-bronchoscopic options for retraction of the orotracheal tube may exist. For patients with poor laryngoscopic views with standard blades, use of a video laryngoscope may provide a superior view [29]. One study described using Doppler ultrasound over the trachea to determine the correct position of the orotracheal tube [30], a technique which is, in our anecdotal experience, less reliable than direct laryngoscopy or bronchoscopy. Laryngeal mask airways have been used successfully instead of orotracheal tubes during PT [31,32], although the relative safety of this technique is debatable [33]. In view of the other advantages, detailed above, it is possible that real-time ultrasound guidance of the needle path will find a role as a routine adjunct, rather than alternative, to standard bronchoscopy-guided PT.

Our study is limited in being only a preliminary demonstration of the feasibility of using real-time ultrasound guidance for tracheal puncture during PT. Also, long term follow-up was not available to detect the incidence of late tracheal stenosis. Larger, randomized studies are required to better define the relative advantages of this technique, appropriate candidates and the safety of US-PT performed without bronchoscopic confirmation of guidewire and cannula placement. We believe our study lays the foundation for future clinical trials.

## Conclusions

Percutaneous tracheostomy can be performed safely using real-time sonographic visualization of the needle path to ensure avoidance of vascular structures and placement of the tracheostomy tube below the first tracheal ring, including in patients with morbid obesity and cervical spine precautions.

## Key messages

• Percutaneous tracheostomy can be performed using real-time ultrasound guidance for visualization of the needle path during tracheal puncture.

• Real-time ultrasound guidance during percutanous tracheostomy can be used to guide placement of the tracheal tube below the first tracheal ring and to avoid vascular structures.

• Real-time ultrasound guidance can facilitate percutanous tracheostomy in patients with morbid obesity and cervical spine precautions.

## Abbreviations

BMI: body mass index; COPD: chronic obstructive pulmonary disease; ICP: intracranial pressure; ICU: intensive care unit; ITD: internal tracheal diameter; PT: percutaneous tracheostomy; SAH: subarachnoid hemorrhage; SD: standard deviation; TBI: traumatic brain injury; US: ultrasound; US-PT: ultrasound guided percutaneous tracheostomy.

## Competing interests

The authors declare that they have no competing interests.

## Authors' contributions

VR conceived of the study, participated in its design and coordination, and drafted the manuscript. JFF, LRR and TLJ participated in the design and coordination of the study, and helped to draft the manuscript. All authors read and approved the final manuscript.
